# Molecular Screening of Black Flies (Diptera: Simuliidae) for Vector‐Borne Zoonotic Pathogens, South Moravia, Czech Republic

**DOI:** 10.1111/zph.70031

**Published:** 2025-11-29

**Authors:** Silvie Šikutová, Kristína Mravcová, Jan Mendel, Oldřich Šebesta, Bohumil Sak, Nikola Holubová, Martin Kváč, Clifton McKee, Peter H. Adler, D. Otranto, Ivo Rudolf

**Affiliations:** ^1^ Institute of Vertebrate Biology Czech Academy of Sciences Brno Czech Republic; ^2^ Biology Centre, Institute of Parasitology Czech Academy of Sciences České Budějovice Czech Republic; ^3^ Faculty of Agriculture and Technology University of South Bohemia in České Budějovice České Budějovice Czech Republic; ^4^ Department of Epidemiology Johns Hopkins Bloomberg School of Public Health Baltimore Maryland USA; ^5^ Department of Plant and Environmental Sciences Clemson University Clemson South Carolina USA; ^6^ Department of Veterinary Medicine University of Bari Bari Italy; ^7^ Department of Veterinary Clinical Sciences City University of Hong Kong Hong Kong China

**Keywords:** arthropod‐borne, black flies, public health, *Simuliidae*, vector‐borne pathogens, vectors

## Abstract

**Introduction:**

Black flies (Simuliidae) are globally distributed blood‐feeding arthropods and vectors of viral, bacterial, and parasitic pathogens to many animal species, including humans. We investigated the occurrence of selected vector‐borne pathogens in black flies in South Moravia, Czech Republic, and evaluated their possible role in the circulation of vector‐borne pathogens.

**Methods:**

A total of 11,600 black flies comprising four species of the genus *Simulium*, namely *Simulium* (*Boophthora*) *erythrocephalum* (De Geer, 1776), *Simulium* (*Wilhelmia*) *lineatum* (Meigen, 1804), *Simulium* (*Wilhelmia*) *balcanicum* (Enderlein, 1924), and *Simulium* (*Wilhelmia*) *turgaicum* (Rubtsov, 1940) were pooled and screened for the following arthropod‐borne pathogens and parasites endemic in Central Europe: viruses (alphaviruses, bunyaviruses and flaviviruses), bacteria ( *Borrelia burgdorferi* sensu lato, *Borrelia miyamotoi* , *Anaplasma phagocytophilum* , *Neoehrlichia mikurensis*, *Bartonella* spp., *Rickettsia* spp., *Francisella tularensis* , *Coxiella burnetii* , and *Brucella* spp.), protista (*Babesia* spp., *Encephalitozoon* spp. and *Enterocytozoon* spp.) and filaria (*Dirofilaria* spp., *Setaria* spp., and *Onchocerca* spp.).

**Results:**

Almost all pools were negative for known arthropod‐borne pathogens and parasites. However, four new *Bartonella* spp. variants were found that share similarity with other bartonellae reported from diverse arthropods and humans. The phylogenetic analysis of *Bartonella* sequences from Czech black flies provides further evidence about an expanding diversity of *Bartonella* lineages in arthropods globally, including hematophagous species (e.g., ticks, mosquitoes, and biting flies) and non‐hematophagous species (e.g., bees and ants). These bartonellae have the potential to cause pathogenic infections in humans who are exposed to arthropods carrying these bacteria.

**Conclusions:**

Summing up, this study provides for the very first time valuable data for characterising the risk to public and veterinary health from black flies and the infections they may carry in Europe. Further testing, however, should include a wider geographic, seasonal, and taxonomic range of black flies.

## Summary

- Black flies (Simuliidae) are globally distributed blood‐feeding arthropods and vectors (both mechanical and biological) of viral, bacterial and parasitic pathogens to many animal species, including humans.
- Four new variants of *Bartonella* spp. have been found in black flies and show similarities to other *Bartonella* reported from various arthropods and humans. These bartonellae have the potential to cause pathogenic infections in humans when exposed to vectors carrying these bacteria.
- This study provides for the first time valuable data to characterise the public and veterinary health risk posed by black flies and the infections they may transmit in Europe.

## 1 Introduction

Simuliids, commonly known as black flies, are blood‐sucking dipterans belonging to the family Simuliidae that occur on all continents except Antarctica. More than 2400 species of black flies have been described and many more species are currently unnamed (P. H. Adler 2025). About 230 simuliid species have been found in Europe (Seitz 2022) in habitats associated with running water, particularly in mountainous regions (Seitz and Adler 2017) but also in dry lowlands (Ruiz‐Arrondo et al. 2023). Indeed, eggs, larvae, and pupae develop in oxygenated running fresh water, with adults being the only terrestrial stage (Adler et al. 2004). Important ecological determinants of species distributions include factors such as elevation, stream size, and water temperature (P. H. Adler 2005; López‐Peña et al. 2022).

Black flies are well recognised as vectors of *Onchocerca volvulus* (Spirurida, Onchocercidae), which causes river blindness in humans. At least 26 *Simulium* species are found in tropical Africa, Latin America and Yemen (Adler and McCreadie 2019), with an estimated 37 million people infected worldwide and 300,000 permanently blind as a result of onchocerciasis in endemic areas, mainly in sub‐Saharan Africa (WHO 2018). Black flies are particularly troublesome for domestic and wild animals (Car et al. 2001), being competent vectors of disease agents of veterinary importance such as avian leucocytozoonosis and filarial nematodes, including those causing bovine onchocerciasis (McCall and Trees 1993). For example, numerous species of black flies are vectors of the blood parasite *Leucocytozoon simondi* in domestic ducks and geese, which causes alterations of erythrocyte membranes resulting in severe anaemia, weight loss, and death (Otranto and Wall 2024). In addition, *Simulium* spp. are implicated in vesicular stomatitis virus (VSV) outbreaks in horses and cattle in the United States (Drolet et al. 2021). Their vectorial competence has been confirmed in experimental studies with colonised and wild‐caught black flies for VS New Jersey Virus (VSNJV) (Mead et al. 1997, 2006). Black flies have also been implicated as vectors of other pathogens, such as *Trypanosoma* spp. to birds, based on epidemiological data and experimental studies (Bennett 1961; Reeves et al. 2007; Votýpka and Svobodová 2004).

Studies of the role of adult simuliids as vectors of disease agents are limited in Europe (Cunze et al. 2024). The full diversity of vector‐borne pathogens transmitted by black flies is insufficiently known because these flies have not been examined as thoroughly as some other blood‐feeding insects, such as mosquitoes and sand flies (Hubálek et al. 2014). There is interest in obtaining a more complete picture of the impact of black flies on public health, as they are significant pests for humans, livestock, and wildlife. For example, simuliids have also been regarded as putative vectors of zoonotic *Onchocerca lupi* (Otranto et al. 2012). Since its first description in a wolf ( *Canis lupus* ) from the Caucasian country of Georgia in 1967 (Rodonaja 1967), *O. lupi* has gained the interest of the scientific community after being reported in humans (Otranto et al. 2011) and increasingly diagnosed in dogs, cats, and humans (Rojas et al. 2021). Some investigations showed that simuliids (Hassan et al. 2015) and mosquitoes (Manoj et al. 2021) tested positive for *O. lupi* in endemic areas of the United States and Portugal, respectively.

Given the relevance of black flies as vectors of emerging vector‐borne pathogens (Cambra‐Pellejà et al. 2020), we investigated the presence of viruses, bacteria, and other pathogens and parasites in black flies in South Moravia, Czech Republic. To our knowledge, this is the first comprehensive field study evaluating the role of black flies in the transmission of viral, bacterial, protist, and filarial pathogens as well as their importance for veterinary and human health in Europe.

## 2 Materials and Methods

### 2.1 Entomological Collections

**FIGURE 1:**
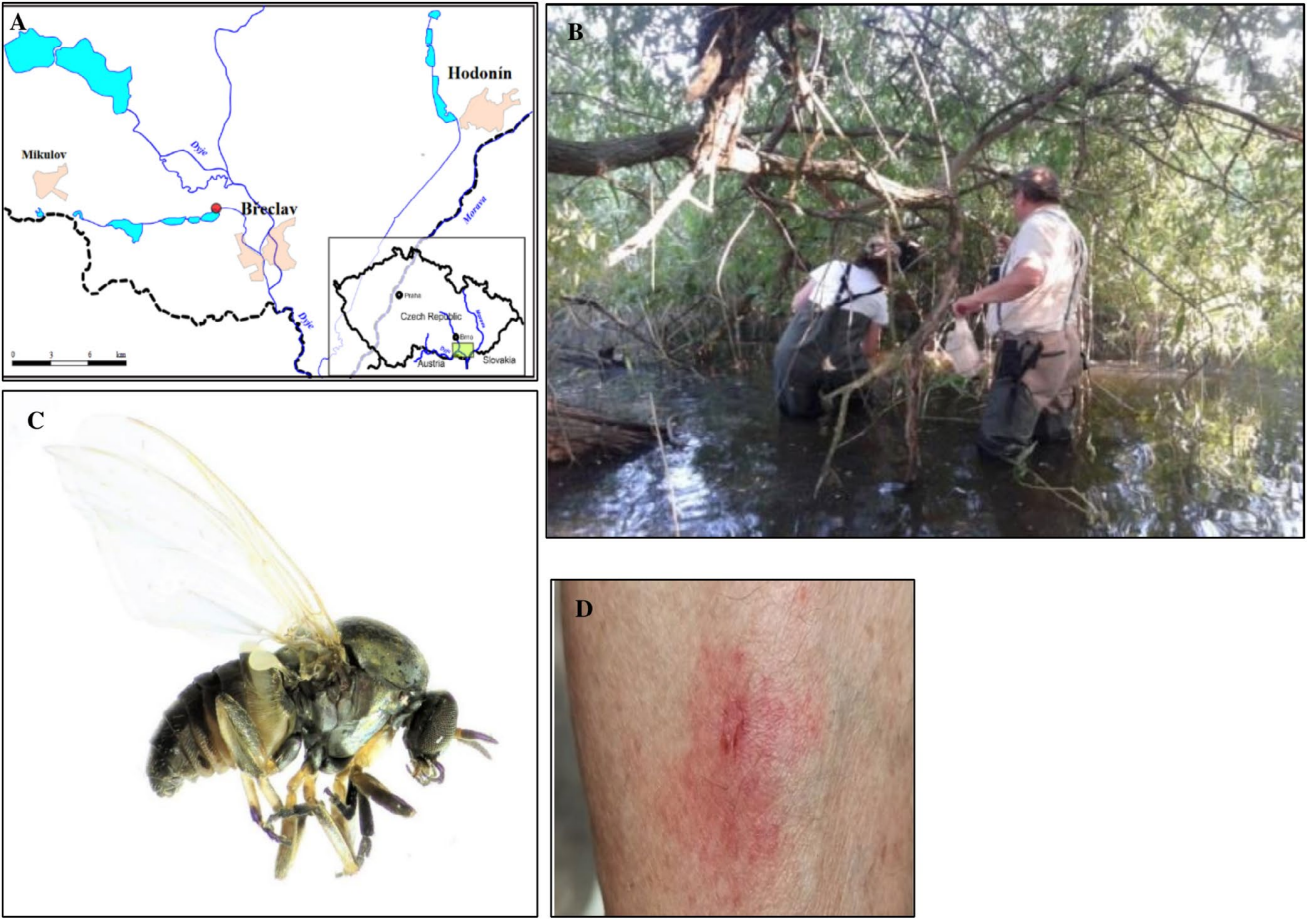
(A) Study site for black fly collections in South Moravia, Czech Republic (red dot); (B) collection of black flies in the field using EVS (Encephalitis Vector Survey) traps; (C) *Simulium* (*Boophthora*) *erythrocephalum* (De Geer, 1776) was the most abundant species in black fly collections; (D) subdermal hematoma caused by painful bites of *Simulium* spp.

Black flies were collected at a fish pond ‘Mlýnský’ in Lednice (48°47′19″ N, 16°49′2″ E; 175 m.a.s.l.) (Figure 1A) during July and August 2023. Specifically, female black flies were trapped using EVS (Encephalitis Vector Survey) traps (BioQuip Products Inc., Rancho Dominiquez, CA, USA) supplemented with dry ice as a source of carbon dioxide and situated in a protected place at a height of 1 m (Figure 1B). Traps were run continuously through the night from 16:00 to 08:00 Central European Summer Time. Captured black flies were transported in closed and chilled containers to the laboratory where they were separated from other insects (e.g., mosquitoes) and stored for further processing in ultralow temperature freezers at −60°C.

### 2.2 Species Determination and Sample Processing

The aim of the study was the evaluation of black flies as possible vectors of vector‐borne pathogens, but not a comprehensive assessment of black fly species diversity. Therefore, a simplified molecular determination was conducted to complement morphological identification of black flies. A total of 100 randomly selected female black flies were identified by sequencing the partial barcoding region of the mitochondrial *cytochrome oxidase subunit* 1 (COX1) gene using previously published primers (Folmer et al. 1994).

For PCR analysis, we used 11,600 female black flies, which were divided into 116 pools (each pool containing 100 individuals, regardless of their taxonomic identity or time period of sampling). Each pool was analysed separately for specific pathogens.

### 2.3 Homogenisation of Black Flies and Nucleic Acid Isolation

Before DNA and RNA isolation, the black flies were surface‐sterilised using 70% ethanol (Top Bio, Czech Republic). Pools were homogenised in 335 μL PBS‐BSA (Phosphate‐Buffered Saline‐Bovine Serum Albumin) using the automated TissueLyzer (Qiagen, Hilden, Germany) with steel beads. From the homogenate, a 100 μL aliquot was used for DNA extraction and 140 μL for RNA extraction. DNA was extracted using the QIAamp DNA Mini Kit (Qiagen, Hilden, Germany), and RNA was extracted using the QIAamp Viral RNA Mini Kit (Qiagen). For the DNA/RNA isolation, the automated QIACube (Qiagen) was used following the protocol for blood and body fluid with manual lysis.

### 2.4 Conventional PCR

For the screening of alpha‐, bunya‐, and flaviviruses, we used conventional PCR with generic primers (Table 1). For PCR reactions, the Qiagen OneStep Kit (Qiagen, Germany) was used. Each reaction tube contained 0.25 μL of both primers (0.1 mM), 1 μL of dNTP (10 mM), 1 μL of enzyme mix, 5 μL of Q‐Solution, 5 μL of 5× Buffer, 7.5 μL of RNase‐free water, and 5 μL of RNA template. RT‐PCR was performed using an Eppendorf EP gradient 96‐well Thermal Cycler (Eppendorf, Hamburg, Germany) under the following conditions: preheating for 30 min at 50°C, initial denaturation for 15 min at 95°C, followed by 40 cycles of 40 s denaturation at 94°C, 50 s annealing at 57°C, and 60 s extension at 72°C, and final extension for 7 min at 72°C. For detection of bacteria, protozoans, microsporidia, and filaria, specific primers, target genes and their lengths are documented in Table 1. PCR protocols were adapted from previously published studies. Amplified products were separated using gel electrophoresis (Mupid One Electrophoresis System, Nippon, Dusseldorf, Germany) with 1.5% agarose gel stained with 12 μL of GelRed (Biotium, San Francisco, CA, USA) and visualised under UV light. Positive samples were reamplified using the same primers, kits, and gels as in the first amplification.

**TABLE 1:** Oligonucleotide primers used for molecular screening of black flies for arthropod‐borne pathogens. Sequences designated {F} are forward primers and those designated {R} are reverse primers.

Taxon	Target gene	Primer sequence	Primer name	Product size (bp)	References
Viruses					
*Togaviridae*	*nsP1*	{F} TCCATGCTAATGCTAGAGCGTTTTCGCA	VIR966	~100	Eshoo et al. (2007)
		{R} TGGCGCACTTCCAATGTCCAGGAT			
*Flaviviridae*	*nsP5*	{F} GTGTCCCAGCCGGCGGTGTCATCAGC	cFD2	~265	Scaramozzino et al. (2001)
		{R} AACATGATGGGRAARAGRGARAA	MAMD		
*Bunyaviridae*	S segment	{F} ATGACTGAGTTGGAGTTTCATGATGTCGC	BSC82C	~251	Kuno et al. (1996)
		{R} TGTTCCTGTTGCCAGGAAAAT	BCS332V		
Bacteria					
*Anaplasma phagocytophilum*	*msp2*	{F} CCAGCGTTTAGCAAGATAAGAG	MSP2f	~334	Eberts et al. (2011)
		{R} GCCCAGTAACATCATAAGC	MSP2r		
‘*Neoehrlichia mikurensis*’	*16S rRNA*	{F} GGCGACTATCTGGCTCAG	micurensis729F	~287	Fertner et al. (2012)
		{R} GCC AAA CTG ACT CTT CCG	micurensis1016R		
*Borrelia burgdorferi*	*rrf‐rrl*	{F} CTGCGAGITCGCGGGAGA		~226–266	Postic et al. (1994)
		{R} TCCTAGGCATTCACCATA			
*Borrelia miyamotoi*	*glpQ*	{F} ATGGGTTCAAACAAAAAGTCACC	glpQ‐BM‐F2	~723	Szekeres et al. (2015)
		{R} CAGGGTCCAATTCCATCAGAATATTGTGCAAC	glpQ‐BM‐R1		
*Francisella tularensis*	*tul4*	{F} GCTGTATCATCATTTAATAAACTGCTG	TUL4‐435	~407	Sjöstedt et al. (1997)
		{R} TTGGGAAGCTTGTATCATGGCACT	TUL‐4‐863		
*Bartonella* spp.	*16S‐23S rRNA*	{F} CTTCAGATGATGATCCCAAGCCTTCTGGCG	BA325s	~300	Diniz et al. (2007)
		{R} GAACCGACGACCCCCTGCTTGCAAAGCA	BA1100as		
*Rickettsia* spp.	*gltA*	{F} GGGGGCCTGCTCACGGCGG	RpCS.877p	~381	Regnery et al. (1991)
		{R} ATTGCAAAAAGTACAGTGAACA	RpCS.1258n		
*Coxiella burnetii*	*com1*	{F} GCTGTTTCTGCCGAACGTAT	CBCOS	~500	Hendrix et al. (1993)
		{R} AGACAACGCGGAGGTTTTTA	CBOE		
*Brucella* spp.	*Bcsp31*	{F} TGGCTCGGTTGCCAATATCAA	Bscp31	~224	Baily et al. (1992)
		{R} CGCGCTTGCCTTTCAGGTCTG			
Protista					
*Babesia* spp.	*18S rRNA*	{F} GTCTTGTAATTGGAATGATGG	BJ1	~400–500	Casati et al. (2006)
		{R} TAGTTTATGGTTAGGACTACG	BN2		
*Enterocytozoon bieneusi*	ITS	{F1} GGTCATAGGGATGAAGAG	EBITS 3	∼390 bp	Buckholt et al. (2002)
		{R1} TTCGAGTTCTTTCGCGCTC	EBITS 4		
		{F2} GCTCTGAATATCTATGGCT	EBITS 1		
		{R2} ATCGCCGACGGATCCAAGTG	EBITS 2.4		
*Encephalitozoon* spp.	ITS	{F1} TGCAGTTAAAATGTCCGTAGT	INT 580 F	∼320 bp	Didier et al. (1995) Katzwinkel‐Wladarsch et al. (1996)
		{R1} TTTCACTCGCCGCTACTCAG	INT 580 R		
		{F2} GGAATTCACACCGCCCGTCVYTAT	MSP 3		
		{R2} CCAAGCTTATGCTTAAGTYMAARGGGT	MSP 4A		
Filaria					
*Onchocerca flexuosa*	*nad5*	{F} TTGGTTGCCTAAGGCTATGG	ND5‐Ov5A‐F	~419	Morales‐Hojas et al. (2006)
		{R} CCCCTAGTAAACAACAAACCACA	ND5OvC‐R		
*Dirofilaria repens*	*COI*	{F} AGTGTTGATGGTCAACCTGAATTA	DR COI‐F1	~200	Rishniw et al. (2006)
		{R} GCCAAAACAGGAACAGATAAAACT	DR COI‐R1		
*Dirofilaria immitis*	*COI*	{F} AGTGTAGAGGGTCAGCCTGAGTTA	DI COI‐F1	~200	Rishniw et al. (2006)
		{R} ACAGGCACTGACAATACCAAT	DI COI‐R1		
*Setaria* spp.	*COI*	{F} TGATTGGTGGTTTTGGTAA	cox1int F	~514	Casiraghi et al. (2001)
		{R} ATAAGTACGAGTATCAATATC	cox1int R		

### 2.5 Sequence Analysis

PCR products were excised from the gel and purified by means of the ZymoClean DNA Gel Recovery Kit (Zymo Research, Irvine, CA, USA). Sanger sequencing of the purified amplicons was performed using a commercial service (SEQme, Dobříš, Czech Republic). All PCR fragments were sequenced bidirectionally to ensure high‐quality reads. DNA sequences were edited and aligned using the Seqman module in Lasergene v. 6.0 (DNASTAR Inc., Madison, WI, USA) and stored in FASTA format. Sequences obtained from black flies were compared to other sequences in the NCBI database (Bethesda, MD, USA), using a BLAST search (http://www.ncbi.nlm.nih.gov/blast). Closely matching sequences were added to a collection of reference sequences for alignment and phylogenetic analysis (see Supporting Information). All sequences were aligned with MAFFT v7 using the E‐INS‐i method recommended for multiple conserved domains and long gaps that are characteristic of alignments (Katoh and Standley 2013). The alignment was visualised in AliView v1.28 (Larsson 2014) and manually trimmed to conserved start and end regions. A maximum likelihood phylogenetic tree was generated in IQ‐TREE v2.1.3, following evolutionary model selection using the Bayesian information criterion (Minh et al. 2020). Branch support was estimated following 1000 bootstrap iterations (Hoang et al. 2018) and the tree was visualised and annotated using the GGTREE package in R (Yu et al. 2017).

## 3 Results

Of the 11,600 black flies collected, four species were identified: *Simulium* (*Boophthora*) *erythrocephalum* (De Geer, 1776) was the most abundant species (11,252 specimens) (Figure 1C), followed by *Simulium* (*Wilhelmia*) *lineatum* (Meigen, 1804) (116 specimens), *Simulium* (*W*.) *balcanicum* (Enderlein, 1924) (116 specimens) and *Simulium* (*W*.) *turgaicum* (Rubtsov, 1940) (116 specimens). Representative COX1 sequences for each vector species discovered were deposited in GenBank under accession numbers PV554191–PV554194.

Of the 116 analysed black fly pools, none tested positive for alphaviruses (including Sindbis virus), bunyaviruses (including Ťahyňa and Batai viruses) and flaviviruses (including tick‐borne encephalitis, West Nile, and Usutu viruses), bacterial pathogens (including *Anaplasma phagocytophilum* , *Neoehrlichia mikurensis*, *Borrelia burgdorferi* sensu lato, *Borrelia miyamotoi* , *Francisella tularensis* , *Rickettsia* spp., *Coxiella burnetii* , and *Brucella* spp.), protozoan pathogens (*Babesia* spp.), microsporidia (*Enterocytozoon* spp. and *Encephalitozoon* spp.), or zoonotic helminths (*Dirofilaria* spp., *Setaria* spp. and *Onchocerca* spp.).

**FIGURE 2:**
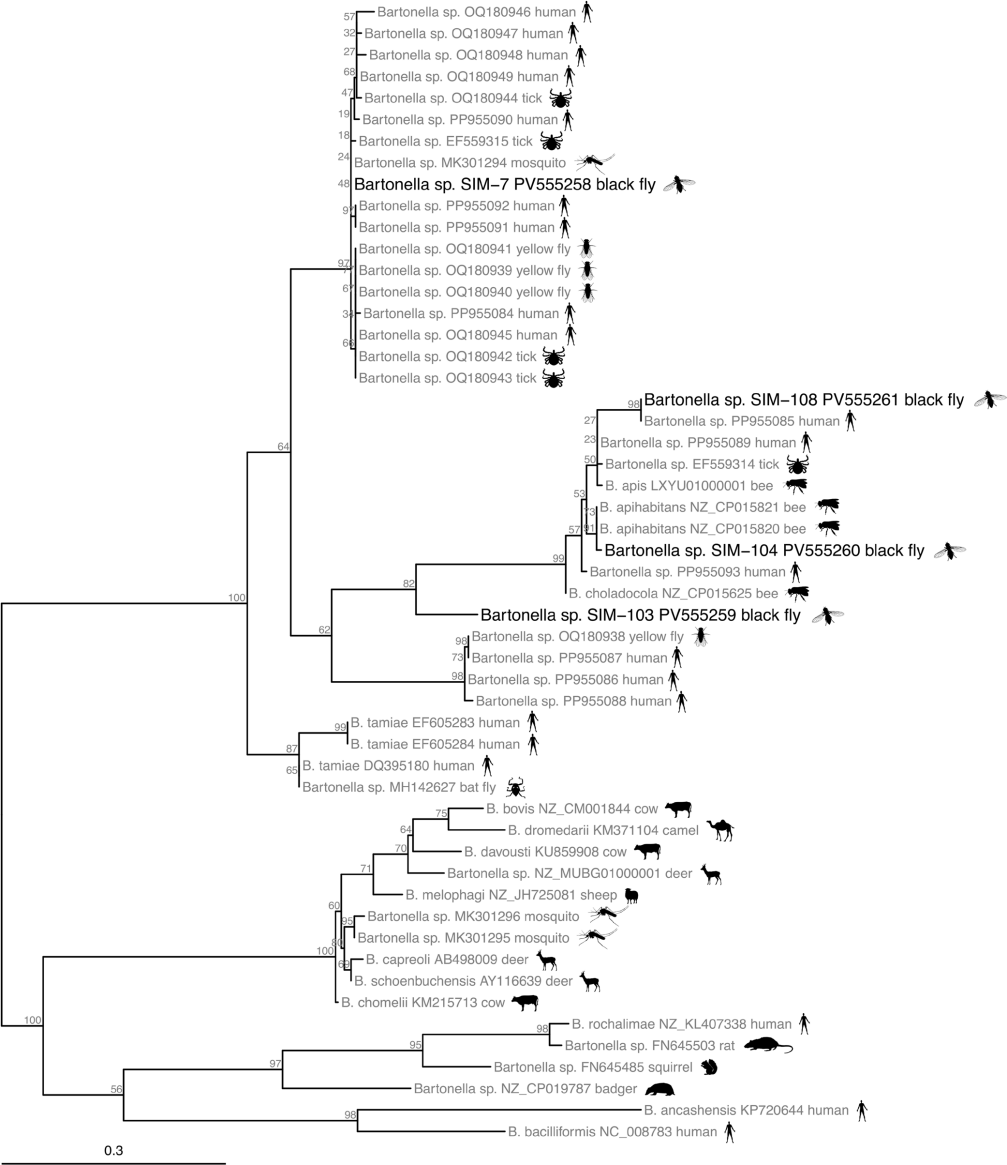
Maximum likelihood phylogenetic tree of Bartonella 16S–23S intergenic spacer (ITS) sequences from Czech black flies and closely matching references. The tree was inferred using a TPM2 + F + I + G4 model in IQ‐TREE from a 932 bp alignment (gaps included) of 53 sequences. Numbers next to nodes show the percent bootstrap support after 1000 replicates. Branch lengths are in units of substitutions per site.

Four pools (designated SIM‐7, SIM‐103, SIM‐104, and SIM‐108) were positive for *Bartonella* spp. All *Bartonella* ITS sequences from black flies were genetically distinct from one another (87.2%–95% sequence identity) and clustered in two separate clades following phylogenetic analysis (Figure 2). Specifically, three sequences fell in a clade containing *Bartonella* species associated with honey bees (*B. apihabitans*, *B. apis*, and *B. choladocola*); a sequence (GenBank accession EF559314) from a tick, *Amblyomma americanum* , collected in North Carolina (Billeter, Miller, et al. 2008); and *Bartonella* sequences (GenBank accessions PP955085, PP955089, and PP955093) detected in dried blood spots from human cases in the United States experiencing chronic Lyme‐like symptoms (Clark and Hartman 2024). One sequence in this clade (SIM‐104, GenBank accession PV555260) was most closely related to *B. apihabitans* strains BBC0178 and BBC0244 isolated from the gut of *Apis mellifera* honeybees in Switzerland (GenBank CP015820 and CP015821; Segers et al. 2017), sharing 99.3% sequence identity with both references according to BLAST. Sequence SIM‐108 (GenBank accession PV555261) shared 99.2% sequence identity with a *Bartonella* variant identified in blood from a human patient with chronic illness in the United States (GenBank PP955085; Clark and Hartman 2024). BLAST identified the closest match for SIM‐103 (GenBank accession PV555259) as *B. tamiae* strains Th307 and Th339 (85.5% sequence identity) isolated from the blood of febrile human patients in Thailand (GenBank EF605283 and EF605284; Kosoy et al. 2008). The phylogenetic analysis showed that SIM‐103 was basal to the same clade containing SIM‐104 and SIM‐108, but the clade was well‐supported overall with 82% bootstrap support (Figure 2).

According to BLAST, the fourth sequence from black flies (SIM‐7, GenBank accession PV555258) shared 100% sequence identity with sequences from *A. vexans* (GenBank MK301294; Rudolf et al. 2020) and from a human patient in the United States (GenBank PP955084; Clark and Hartman 2024). SIM‐7 formed a well‐supported clade (97% bootstrap support) with numerous other *Bartonella* sequences in arthropods, including *Amblyomma americanum* ticks from North Carolina and Florida (GenBank accessions EF559314 and OQ180942–OQ180944), *Diachlorus ferrugatus* yellow flies (Tabanidae) from Florida (GenBank accessions OQ180939–OQ180941), and human patients with chronic illness in the United States (Billeter, Miller, et al. 2008; Rudolf et al. 2020; Clark and Villegas Nunez 2023; Clark and Hartman 2024). This clade contained sequences (GenBank accessions OQ180945–OQ180949) detected over several years in a Florida resident suffering from chronic relapsing symptoms, including lymphadenopathy, malaise, headache, somnolence, sleep disturbance, and occasional brain fog and stiff neck; sequences from yellow flies and ticks were obtained from sampling at the patient's residence (Clark and Villegas Nunez 2023). Additional sequences from human patients experiencing chronic Lyme‐like illness in the United States fell into this clade (GenBank accessions PP955084 and PP955090–PP955092).

## 4 Discussion

Only about 1.5% of the black fly species worldwide are currently known to be competent vectors of human pathogens. We evaluated the possible role of black flies in the transmission of a selected set of viral, bacterial, protozoan and filarial vector‐borne pathogens and parasites circulating in the region of South Moravia, Czech Republic. The species of black flies identified in our study are abundant mammalophilic pests throughout much of Europe, commonly feeding on livestock (Pápay et al. 1971; Werner and Adler 2005). *Simulium erythrocephalum* and *S. turgaicum* are also highly aggressive and bite humans (Ignjatović‐Ćupina et al. 2006; Sariözkan et al. 2014), setting the scene for the potential transfer of pathogens and parasites from domestic animals to humans. The wild hosts of these black flies are poorly known but would include an expansive pool of blood hosts from which pathogens and parasites could potentially be transferred to humans.

The phylogenetic analysis of *Bartonella* sequences from Czech black flies contributes to identifying and expanding our knowledge about the diversity of *Bartonella* lineages in arthropods globally, including hematophagous species (e.g., ticks and biting flies) as well as non‐hematophagous species (e.g., bees and ants). Certain *Bartonella* species (e.g., *B. henselae* , *B. bovis* , *B. elizabethae* ) are known to infect a diverse set of mammalian hosts and can have a nearly global distribution (Bai et al. 2013; Kosoy and Bai 2019; Zarea et al. 2023), and numerous zoonotic *Bartonella* have been shown to cause incidental infections in humans (Cheslock and Embers 2019). The newly identified *Bartonella* lineages from this study are separate from the group of *Bartonella* species that infect mammals and may have various ecological roles, potentially as commensal or mutualistic symbionts in their arthropod hosts (Kešnerová et al. 2016; Segers et al. 2017; Bisch et al. 2018; Liu et al. 2022; Xiong et al. 2024). However, these bartonellae have a similar potential to cause illness in incidentally infected humans that are exposed to arthropods carrying them, as evidenced by the human cases of *B. tamiae* in Thailand (Kosoy et al. 2008). *Bartonella tamiae* was later detected in ectoparasitic mites from rodents in Thailand (Kabeya et al. 2010), bat ticks in Algeria (Leulmi et al. 2016), and bat flies in Nigeria (Bai et al. 2018). Furthermore, human cases reporting chronic physical and neurological symptoms from Florida and several other U.S. states showed detections of *Bartonella* variants related to *B. tamiae* and other *Bartonella* species found in arthropods (e.g., *B. apis*, *B. apihabitans*, and *B. choladocola*) in the patients' blood (Clark and Villegas Nunez 2023; Clark and Hartman 2024). In the case of the Florida patient, *Bartonella* variants identical to those found in their blood were identified in ticks and yellow flies collected from their property (Clark and Villegas Nunez 2023). This suggests that incidental infections of *Bartonella* following transmission from biting flies may occur, though more study will be needed to understand the frequency of such transmission and define the clinical syndrome associated with infection in humans or other host animals. While the risk of these infections is difficult to ascertain from these preliminary studies, increased surveillance and development of methods for detection of these *Bartonella* spp. clades may reveal more cases and identify risk factors for infection. Additional genetic information from these *Bartonella* clades in arthropods will shed light on the evolutionary history of the genus and its ecological plasticity.

In addition to their role as vectors of pathogens, black flies are of medical concern because, when blood feeding, they may induce subdermal hematomas (Figure 1D). In addition, due to their buccal apparatus, they are pool feeders causing painful bites, some of them (e.g., *S. erythrocephalum* and *S. turgaicum*) inducing local skin lesions, painful erythema, allergic reactions, and respiratory and circulatory symptoms (Beaucournu‐Saguez et al. 1993; Sariözkan et al. 2014; Akhoundi et al. 2020), elicited by components (e.g., anticoagulant proteins) of the saliva (Cupp and Cupp 1997).

Our study suggests that black flies, as possible vectors of a selection of zoonotic pathogens, do not pose a major risk to public health, at least in this sampled region of the Czech Republic. However, there are a few caveats that we must point out. First, collection efforts should be performed in other geographical areas of the region and during different periods of the year as early season, univoltine species could be missed during the collection period (i.e., July–August). The greatest number of attacks on humans by black flies in the affected area is from May to July (Sitarz et al. 2022). Second, our detection of *Bartonella* was limited to bacterial DNA, which provides no information on whether bartonellae were alive and replicating in black flies. More investigations—including detection of mRNA or culturable bacteria in black flies and incidental hosts (Kosoy et al. 2016), as well as vector competence studies (Bown et al. 2004; Billeter, Levy, et al. 2008; Wu et al. 2022) – are required to identify the role that black flies play in the maintenance of these bacteria in nature. Black flies may serve as potential vectors of these bacteria to some unidentified vertebrate host or as definitive hosts of mutualistic or commensal bacteria that may only infect vertebrates through incidental exposure via blood feeding. Regarding the first possibility, black flies typically must blood‐feed at least twice to serve as vectors, once to acquire pathogens and parasites and again to transfer them (Crosskey 1990). Blood meal analysis, including genotyping of host DNA, would also be helpful for evaluating the importance of blood feeding in the maintenance of these *Bartonella* lineages among black flies and other vertebrate hosts (Khanzadeh et al. 2020; Gomontean et al. 2024). Under the above circumstances, we do not know the proportion of collected insects that had completed at least one gonotrophic cycle being potentially infective. On the other hand, some animal pathogens can be transmitted vertically within arthropods (de Bruin et al. 2015), whereas others might be transmitted horizontally at flowers (McArt et al. 2014; Proesmans et al. 2021), a little‐explored infection pathway for black flies. Adult black flies acquire sugars for flight and other metabolic activities by feeding from floral nectar and hemipteran honeydew (Burgin and Hunter 1997), potentially exposing them to pathogens that could then be incidentally transmitted to blood hosts. Those listed above represent only a part of the many difficulties in studying these insects and their role as vectors of pathogens. Other hindrances in studying black flies in the field are represented by insect collection and breeding them under laboratory conditions.

Overall, our study is the most comprehensive survey of potential vector‐borne pathogens in black flies. The study highlights the first discovery of bartonellae in black flies and the need to understand the paths of infectivity, simuliid–pathogen dynamics, and risks to humans and their animals. While the overall results should allay immediate concerns of simuliid‐borne disease risks for humans and domesticated animals in South Moravia, we caution that the public health and medical–veterinary communities must remain vigilant to possible threats. We emphasise that less than 10% of the 45 species of black flies known from the Czech Republic were screened at only one point in time and space in a country of diverse landscapes of nearly 79,000 km^2^. Additional studies will be needed to survey a wider diversity of simuliid species in this region, as well as other dipteran vectors like midges and sandflies (Ceratopogonidae) or *Stomoxys* flies (Muscidae) that carry viruses and other parasites with veterinary and public health relevance (e.g., bluetongue, lumpy skin disease, and Oropouche fever viruses) (Baldacchino et al. 2013; Kampen and Werner 2023).

Summing up, this study provides novel and valuable data for characterising the risk to public and veterinary health from black flies and the infections they may carry in Europe.

## Author Contributions

I.R. conceived and designed the study. S.Š., K.M. and O.Š. collected black flies in the field. K.M., S.Š. and O.Š. identified black flies. S.Š., K.M., B.S. and N.H. performed laboratory analyses and interpreted results. J.M. and C.M. performed sequencing and bioinformatic analysis. I.R., P.H.A., D.O., C.M. and M.K. coordinated the study and drafted the manuscript. All authors reviewed and approved the final version of the manuscript.

## Funding

The study was financially supported by the Ministry of Health of the Czech Republic (Reg. No. NU21‐05‐00143).

## Ethics Statement

The authors have nothing to report.

## Consent

The authors have nothing to report.

## Conflicts of Interest

The authors declare no conflicts of interest.

## Supporting information


**Data S1:** Biological source information and GenBank accession numbers for all sequences used in alignment and phylogenetic analysis.

## Data Availability

The data that support the findings of this study are available from the corresponding author upon reasonable request.
